# Saving patients one stitch at a time?

**DOI:** 10.1016/j.igie.2023.04.002

**Published:** 2023-05-12

**Authors:** Chas McKhann, Linda S. Lee

## Editor’s Introduction

For several decades, therapeutic endoscopy focused on ERCP supplemented with diagnostic EUS. However, over the past couple of decades, a dizzying array of advances has propelled the field into the realm of endoscopic surgery. One key device responsible for this revolution in our field is the endoscopic suturing device. The first endoscopic suturing device actually dates back to 1994 when the Endocinch (CR Bard Inc, Murray Hill, NJ, USA), developed by Dr Paul Swain, was first used in humans in the United Kingdom and was approved by the U.S. Food and Drug Administration (FDA) in the United States in 2000.^1^ The Endocinch was primarily used for treatment of GERD in thousands of patients but was limited by a short-lived clinical response, likely because of the lack of full-thickness suturing.^2^ It was first used for bariatric endoscopy in 2003 by Dr Christopher Thompson, who performed transoral outlet reduction in gastric bypass patients.^3^ Numerous devices ensued, including the NDO Plicator (NDO Surgical, Mansfield, Mass, USA), EsophyX (EndoGastric Solutions, Redmond, Wash, USA), Endoscopic Suturing Device (Wilson Cook, Winston-Salem, NC, USA), g-Prox (USGI, San Clemente, Calif, USA), and Eagle Claw (Olympus Medical Systems Corp, Tokyo, Japan), the predecessor to the OverStitch device (Apollo Endosurgery, Austin, Tex, USA).

An obvious application of endoscopic suturing is to close large defects, including perforations, fistulae, and leaks, on which the first reports of the OverStitch device focused.^4^^,^^5^ These case reports described persistent gastrocutaneous fistulae after removal of a percutaneous gastrostomy tube despite the use of clips, including over-the-scope clips, and subsequent successful closure using the OverStitch endoscopic suturing device (Apollo Endosurgery). Dr Thompson’s group then reported a novel use to close refractory large marginal ulcers, and Drs Kantsevoy and Swanstrom’s groups described proximal stent fixation of esophageal stents placed for benign and malignant indications.^6^, ^7^, ^8^ As endoscopic submucosal dissection and third-space endoscopy with submucosal tunneling for various myotomy procedures and resection have expanded, endoscopic suturing has proven useful for closing the defects from these procedures. Of course, the field of bariatric endoscopy arose from the birth of endoscopic suturing devices as mentioned earlier.

Currently, 3 devices have received FDA clearance for tissue approximation: the OverStitch Endoscopic Suturing System (Apollo Endosurgery), Incisionless Operating Platform (USGI), and Endomina plication system (Endo Tools Therapeutics, Gosselies, Belgium). The OverStitch device is the only one with FDA De Novo Marketing Authorization on July 13, 2022 for endoscopic sleeve gastroplasty (ESG) and endoscopic bariatric revision. Thus, it is my pleasure to have a conversation with Chas McKhann, former President and Chief Executive Officer (CEO) of Apollo Endosurgery and current advisor to Boston Scientific. Before Apollo, Mr McKhann served as CEO or Chief Commercial Officer roles in 4 medical device companies including Torax Medical and Intersect ENT. Previously, he served in senior leadership roles at Boston Scientific Corporation and Cordis, a Johnson & Johnson company. Early in his career, Mr McKhann was a strategy consultant at McKinsey & Company. Mr McKhann holds a bachelor’s degree and a Master of Business Administration from Stanford University.

**Linda Lee (LL): Would you discuss the history behind how Apollo was founded? Also, how and why did Apollo decide to pursue developing its marquee OverStitch Endoscopic Suturing system all those years ago?**

**Figure 1:**
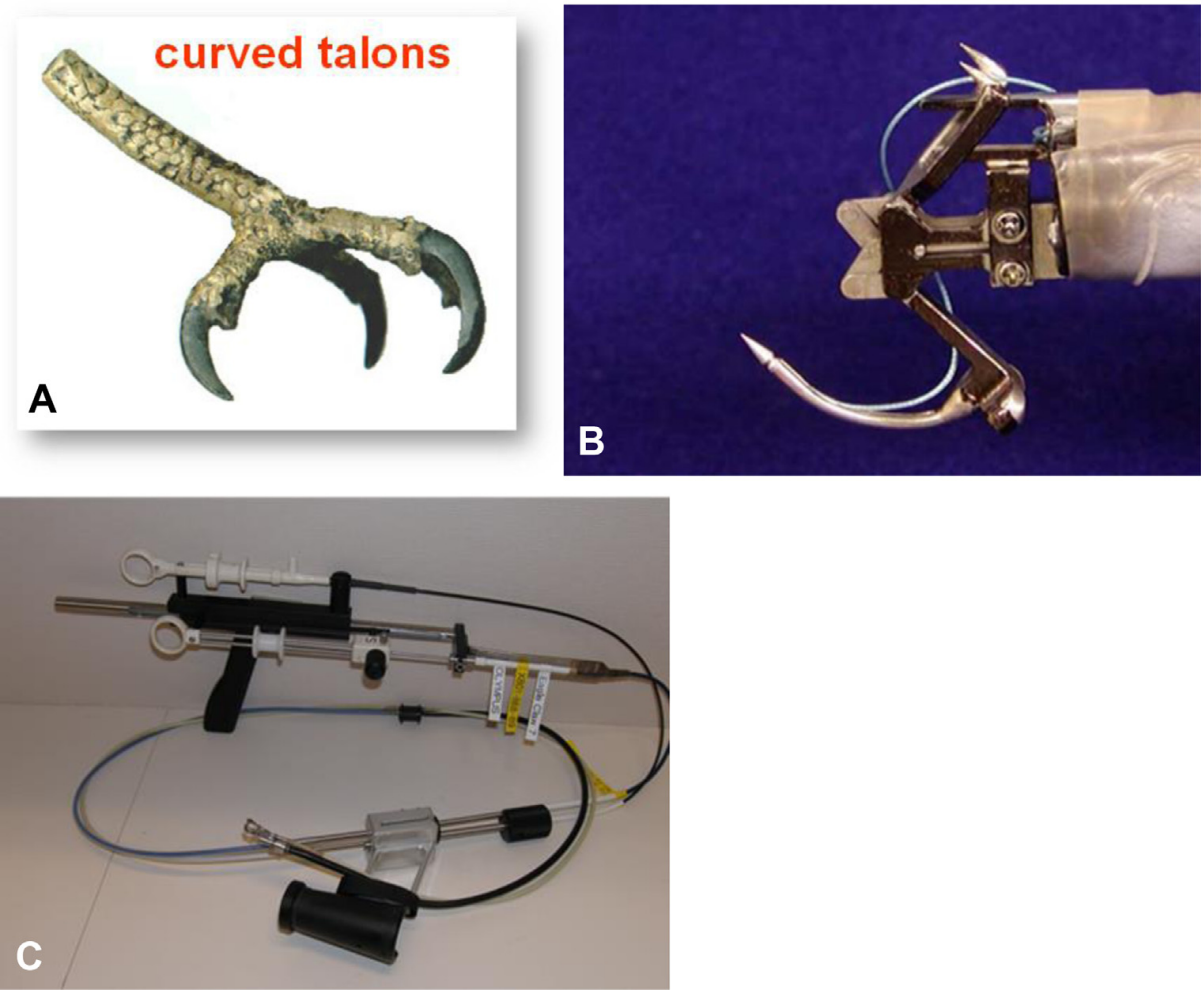
**A** and **B,** The Eagle Claw suturing device with curved needle arm, transferable needle anchor, and reciprocating tissue-capturing “claws.” **C,** The complexity of device is apparent.

**Chas McKhann (CM):** In 1998, a visionary group of interventional endoscopists formed the Apollo Group to develop novel approaches to interventional endoscopy. The original members of the Apollo group were Dr Sidney Chung, Dr Peter Cotton, Dr Christopher Gostout, Dr Rob Hawes, Dr Tony Kalloo, Dr Sergey Kantsevoy, Dr Jay Pasricha, and Dr Ted Truss. In the early days, the Apollo Group developed a series of tools to facilitate natural orifice transluminal endoscopic surgery (NOTES) procedures. At the conclusion of a NOTES procedure, a physician needed a reliable and easy-to-use endoscopic suturing device to close the incision using full-thickness suturing. The mechanism of endoscopic suturing was initially proposed by Sidney Chung from the Chinese University of Hong Kong, who mandated the device mimic an eagle’s claw using a curved needle arm. During this initial period, the Apollo Group was supported by Olympus whose engineers helped construct the forerunner of OverStitch, the Eagle Claw (Fig. 1).

In 2006, members of the Apollo Group founded a company, Apollo Endosurgery, and recruited Dennis McWilliams to serve as CEO. Apollo was a classic startup comprised of Dennis plus a few engineers working out of office space provided by one of the company’s early investors in downtown Austin (before this, the team met in a local coffee shop). Also during this time, a joint committee comprising American Society for Gastrointestinal Endoscopy and Society of American Gastrointestinal and Endoscopic Surgeons members, named NOSCAR, was established, and the initial NOSCAR meetings included all founding members of the Apollo Group as well as other innovators, such as Dr Christopher Thompson and Dr Lee Swanstrom. From its beginning, NOSCAR has emphasized the critical role of collaboration between gastroenterologists and surgeons in the development of endoscopic procedures and technologies.

**LL: How long did it take from the idea to developing the first product that was brought to market? Would you discuss the steps along this journey of manufacturing this device?**

**CM:** It took more than 10 years from the initial conceptual idea by the Apollo Group to the first endoscopic suturing case in a human using OverStitch Gen 1. The initial Eagle Claw device was inconsistent in satisfying the greatest challenge, providing full-thickness stitches to ensure durability, and raised concerns regarding the ability to safely close intentional NOTES gastrotomies. The gastric wall proved to be a challenge to consistently puncture.

**Figure 2:**
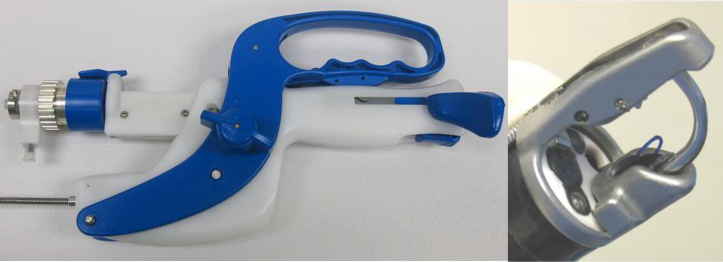
First-generation commercially available “Gen 1” OverStitch device. The large bulky handle resulted in a complex series of steps needed to suture.

Building on the original concepts of the Eagle Claw, the Apollo engineering team, led by Don Jones, Vlad Mitelberg, and Brett Naglreiter, developed a gear-driven suture platform with a curved needle able to perform interrupted and running stitches and was reloadable with a new suture without having to remove the device from the patient. The first commercially available Overstitch device (“Gen 1”) was introduced in 2009 (Fig. 2). The first case with the Gen 1 device in 2009 was performed by Dr Gostout at the Mayo Clinic and involved closure of a persistent esophageal fistula with suturing of a fully covered stent over the sutured closure. Unfortunately, this first-generation device was bulky and complex to operate. A master endoscopist like Sergey Kantsevoy could make it work, but the product was not ready for widespread adoption. It also was far too costly to manufacture and would not have been a viable commercial product.

**Figure 3:**
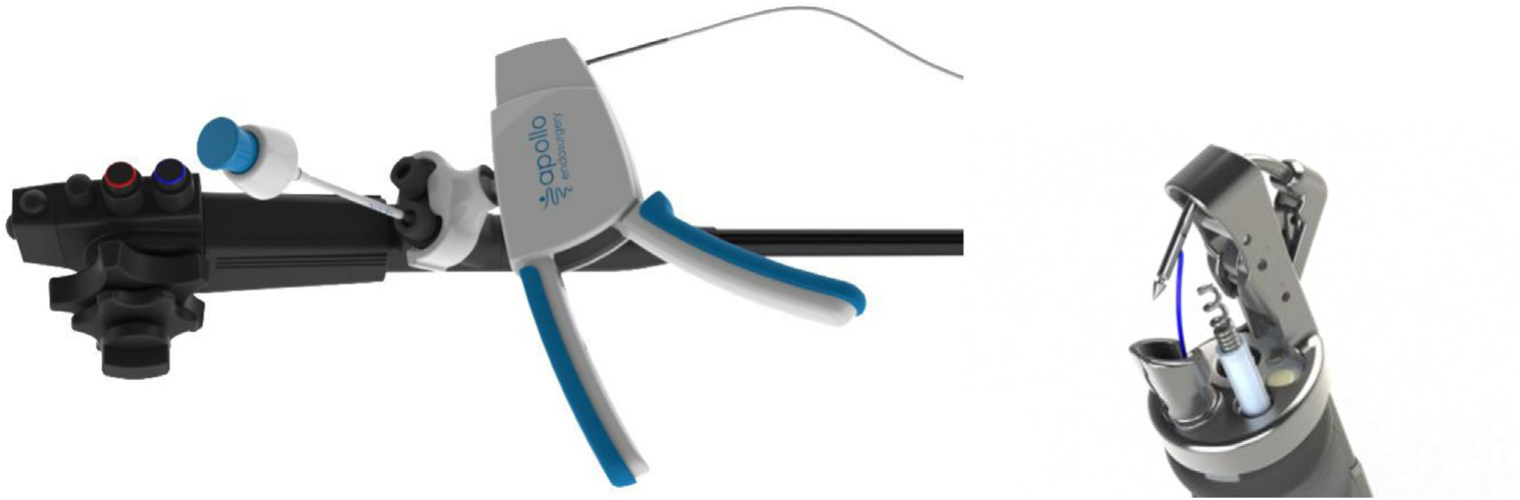
The Gen 2 two-channel OverStitch device with a tissue-capturing helix.

The Gen 2 device (Fig. 3), which is the same dual-channel version of the OverStitch used today, was designed to be easier to operate, have a shorter learning curve, and be less expensive to manufacture. This version of OverStitch was cleared by the FDA and first commercialized in 2011.

**LL: What was the biggest challenge during this process?**

**CM:** The company encountered numerous challenges in its early years. The first was regulatory. The company’s original focus on NOTES procedures was predicated on the assumption that all the devices required to do NOTES procedures could be approved through the FDA’s “510k” regulatory process. Unfortunately, Johnson & Johnson’s Ethicon Endosurgery group had approached the FDA regarding its NOTES tools, and the FDA stated that these tools would need to go through the much more time-consuming and expensive premarket approval process. This decision fundamentally changed the amount of time and investment required to develop new tools for NOTES and resulted in a massive collapse in research and development (R&D) investment and activity for NOTES-related applications.

The other major challenge facing the company was financial. After a strong start and good support from the venture capital community, Apollo needed to raise additional “Series B” capital during 2008. The downturn of the global financial system in late 2008 led to enormous challenges across the innovation ecosystem. The company was able to squeak by on bridge and grant financing and several painful rounds of layoffs.

**LL: What modifications have been made to the device over the years since the original device? It sounds pretty simple to make the device compatible with scopes from different companies, but I imagine this was not easy to accomplish. Would you talk about how this was done?**

**Figure 4:**
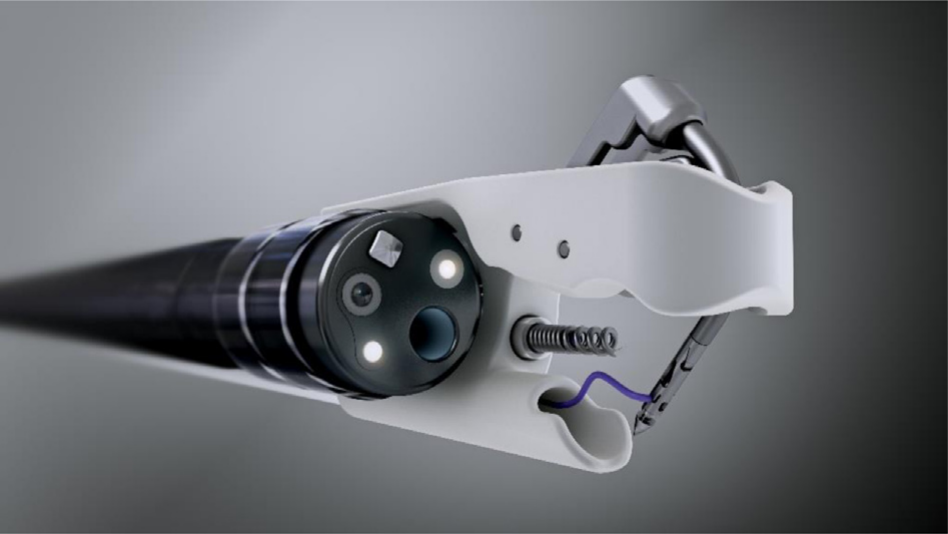
The OverStitch Sx device with side-mounted features that allow universal adaption to single-channel endoscopes.

**CM:** Development of a single-channel version, the OverStitch Sx device (Fig. 4), required placing the channels and cables outside the endoscope insertion tube with the suturing device fixed to the side of the endoscope tip as opposed to the Gen 2 device, which is mounted directly over the endoscope tip. In addition, a key design requirement was that this arrangement provided haptics that would be easy to use and would appeal to the endoscopist.

One of the main challenges in developing a single-channel version of the OverStitch device was to redesign the endcap and develop a reliable attachment solution to mount the suture mechanism to the outside of any single-channel endoscope in a repeatable and intuitive manner to ensure the needle trajectory was always in the desired orientation. Although many single-channel scopes look the same, small differences in diameters of channels, orientation of light sources and lenses, and even reprocessing effects can have significant impacts when trying to precisely line up the needle capture system. The attachment solution also had to be very low profile to ensure compatibility with existing endoscopic access systems. Achieving a low cost of goods target was also challenging because a lot more features like the working channels for tissue helix and anchor exchange had to be added to the suturing device, which added more complexity. It took 5 years to overcome these engineering challenges, and the OverStitch Sx device was launched in 2018.

**LL: Would you discuss the FDA approval process and implications of the FDA De Novo Marketing Authorization for ESG and endoscopic bariatric revision in July 2022?**

**CM:** Initially, Apollo was mainly focused on the U.S. market. The Gen 1 and later the Gen 2 versions of the OverStitch device were cleared through the 510(k) process. OverStitch was first approved outside the United States in 2012, a year after the U.S. launch of the Gen 2 device. The approved indications in both jurisdictions were limited to apposition of soft tissue and endoscopic placement of suture(s). Although physicians could use OverStitch in a wide range of cases, many applications such as ESG were considered “off-label” by regulatory authorities.

From the early days of the Apollo Group, innovators envisioned using a suturing device for a gastric weight loss procedure, and in April 2012, the first ESG procedure was performed by Dr Christopher Thompson and Dr Rob Hawes in India.^9^^,^^10^ Since then, more than 25,000 ESG procedures have been performed worldwide using OverStitch. More than 6500 patients have been studied in ESG published clinical studies, and more than 200 publications have been published on ESG and bariatric revision procedures using OverStitch.^11^, ^12^, ^13^, ^14^, ^15^, ^16^

However, prospective level 1 evidence is required to achieve regulatory clearance in support of a specific weight loss claim and to thereby achieve wider market adoption. Apollo Endosurgery sponsored the MERIT study, a prospective randomized controlled trial of ESG compared with diet and exercise, which was initiated in 2017 and completed in 2021. After successful completion of the study with a 2-year follow-up, results from the MERIT trial were published in *The Lancet* in 2022.^17^

Because there is no existing predicate for a device for an ESG procedure, the FDA required Apollo to follow the De Novo regulatory clearance process versus a standard 510(k). Using a regulatory submission that was based on the MERIT study outcomes and data collected from extensive real-world experience, Apollo Endosurgery applied for FDA regulatory clearance for 2 new products, Apollo ESG and Apollo REVISE, and received marketing authorization in July 2022.

**LL: How have you ensured that physicians using the OverStitch device are properly trained and able to troubleshoot when there are problems to avoid major adverse events from occurring?**

**CM:** The team at Apollo has been attentive to providing the necessary opportunities to educate and train physicians using our devices for appropriate applications. Hands-on training and a robust medical education program have been key to successfully bringing endoluminal suturing into clinical practice. Considerable oversight is applied to content and accuracy of printed materials, such as the instructions for use documents, PowerPoint (Microsoft, Redmond, Wash, USA) presentations, and training models.

The company provides full-day training courses that involve a combination of didactic instruction and extensive hands-on experience at locations including our headquarters in Austin, Texas, at major GI and surgical meetings, and a new series of endobariatric courses held at the American Society for Gastrointestinal Endoscopy ITT Center in Chicago. We also use a mobile learning center, which is an 18-wheel truck that includes 5 endoscopy stations to allow in-person, hands-on training for our products throughout the United States.

**LL: Would you discuss your latest X-Tack device and what led to development of this device?**

**Figure 5:**
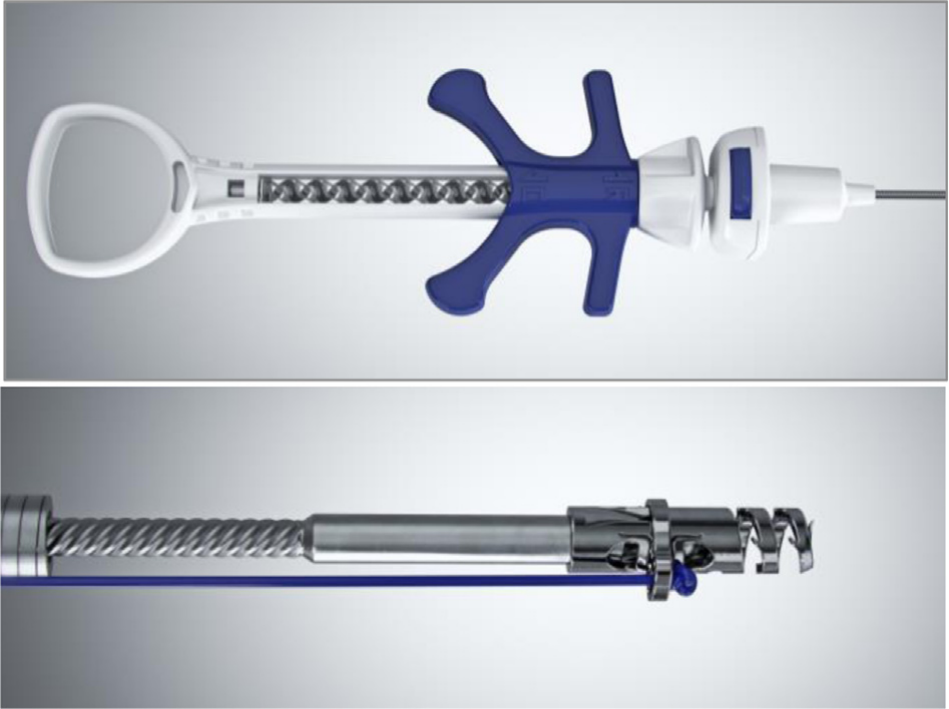
X-Tack Helix tack and Persian Drill handle.

**CM:** The X-Tack device (Fig. 5) was developed based on customer feedback asking for new tools to provide effective closure of defects in the colon. OverStitch had been created to offer foregut suturing during the birth of NOTES and in particular needing to ensure full-thickness suture passage through the thicker wall of the stomach. The use of OverStitch in the colon requires the use of a specialized overtube, but also this tissue is thinner and more delicate than that found in the stomach. The ongoing attention in publications, presentations at major meetings, and clinical practice advocating prophylactic closure of polypectomy sites, along with EMR and endoscopic submucosal dissection defects, in the colon to prevent delayed bleeding and perforation, especially in recognized high-risk groups, drove the X-Tack project into action.

Although through-the-scope clips and even over-the-scope clip devices are used for some of these purposes, there are technical limitations to each type of device. These limitations restrict success in the closure of large, wide, and irregularly shaped defects, particularly in difficult to reach anatomies. The Apollo team, working with clinical advisers such as Dr Andrew Storm, confirmed that we needed to provide a suturing-like through-the-scope experience, allow universal adaptability to any endoscope by being compatible with a 2.8-mm or larger working channel, and maintain simplicity of operation.

After a series of iterations on many different functional prototypes, our engineering team, led by Tom Neudeck, recognized the value of the helix in tissue capture. Building on our understanding on the biomechanics of helix engagement of soft tissue, a separable helical coil from a deployment catheter with attached suture became the working concept. In addition, X-Tack is the sole endoscopic therapy device that incorporates the idea of using the Persian drill concept, which translates linear motion into rotation, to allow screwing the HeliX Tack into the gut wall in a very precise and repeatable manner. X-Tack was developed during the height of the COVID (coronavirus disease 2019) pandemic, and after overcoming some massive hurdles associated with product development during this time, X-Tack was cleared by the FDA for use in the United States in December of 2020.

**LL: Apollo seems to have a strong leadership position in the field of endoscopic suturing. Why do you believe this has been the case all these years?**

**CM:** First, a full-thickness suturing device must overcome many significant engineering challenges. The primary function of the device was to provide consistent full-thickness bites. This required the delivery of sufficient force to drive the needle arm through tough tissues such as the gastric wall. Developing a miniaturized gear-driven suturing mechanism that securely attaches to the tip of an endoscope and designing a robust needle exchange mechanism that is compatible with a 3.7-mm endoscope channel were significant engineering hurdles that the Apollo engineering team managed to overcome. The other complex design challenge was to develop a device that lets the physician secure and terminate the suture because it is not possible to tie a surgical suture knot at the end of the endoscope.

Second, the funding environment for novel technologies in therapeutic endoscopy has been difficult. Over most of the past 10 to 15 years, the advanced therapeutic endoscopy market has been a nascent market, and procedure volumes have been relatively low. There are also significant technical risks and market adoption risks associated with these devices, and these risks scared off many prospective investors. As a result, it has been challenging for Apollo and other companies in this field to attract the required investment for R&D and market development. Third, intellectual property that the Apollo Group secured specifically around using a curved needle for intraluminal suturing and Apollo Endosurgery’s intellectual property around a detachable endcap containing a suturing mechanism, as well as other patented features of the technology that have been developed over time, have also helped protect OverStitch’s competitive position.

**LL: Would you discuss the thought process behind deciding to take a company public and the implications of this for the company?**

**CM:** Developing and commercializing novel medical technology is difficult … and very expensive. In total, the development of OverStitch, including R&D activities, sponsoring the MERIT study and commercial activities, is estimated to have cost well in excess of $100 million. A robust triumvirate composed of clinicians, engineers, and financiers is typically required to transform a compelling vision into a successful company. For Apollo, the financial vision came from PTV Sciences, including Dennis McWilliams, who was an entrepreneur in residence at the time he founded Apollo, and Rick Anderson, who had joined PTV after previously serving as a senior executive at Johnson & Johnson. They were fully enrolled in the vision for endoscopic suturing to transform therapeutic endoscopy and were able to recruit other venture capital firms such as HIG Capital, Novo Ventures, and CPMG to provide funding for the company, and together they helped Apollo navigate some extremely challenging times that nearly sunk the company.

In 2016, Apollo underwent a process to become a publicly traded company through a reverse merger, which allowed access to a larger and more diverse investor group. Money raised during this time supported the launch of the Orbera intragastric balloon and completion of the MERIT study. More recently, in 2021, Apollo completed an additional equity raise of more than $75 million to strengthen the company’s balance sheet and allow the company to further invest in activities, including expanding our sales force, generating additional clinical evidence, training new physicians, and advancing our R&D pipeline.

**LL: When you joined Apollo as their CEO in 2021, what were your top 3 priorities?**

**CM:** As I conducted my research to decide whether to take on the role of CEO at Apollo, it was clear that the company offered innovative technologies that can have a meaningful impact in clinical practice. However, Apollo had been hit hard during the COVID pandemic and was under-investing in key initiatives to successfully build adoption of these products.

To address this, my 3 biggest priorities have been as follows:

1. *Revitalize the organization:* We recruited new leadership in areas such as sales, marketing, and health economics and reimbursement to complement an already strong R&D and clinical team. We also focused on building a strong corporate culture that is based on 5 core values: patient centric, customer focused, innovative, passionate, and caring.
2. *Invest in training and market development:* We have added new programs to train more physicians on the appropriate use of OverStitch, X-Tack, and Orbera and have increased the adoption of all 3 product lines. As I mentioned previously, we raised more than $75 million to support these efforts.
3. *Develop the endobariatric opportunity:* In the last year, the MERIT study was published in *The Lancet*, and we received FDA marketing authorization for Apollo ESG and Apollo REVISE. Going forward, we are working with leading GI and surgical societies to implement new endobariatric training programs, supporting postapproval clinical research, and engaging in initiatives to support reimbursement coding, coverage, and payment for endobariatric procedures.

**LL: Congratulations on the acquisition of Apollo by Boston Scientific. Are you able to discuss how this came about, the process, and how long the process has been ongoing?**

**CM:** For many years, there has been considerable interest from large medical device companies in Apollo Endosurgery’s products, especially OverStitch. As Apollo’s CEO, my focus has been on building a successful, high-growth company that has the financial resources to succeed as a stand-alone company.

Boston Scientific had other plans. Discussions between Boston Scientific and Apollo originated more than 10 years ago. The team at Boston Scientific is familiar with our products and markets, and they have been tracking the significant positive momentum that the company has experienced in recent years. In the second half of 2022, acquisition discussions with Boston Scientific progressed rapidly, culminating in a deal announcement in November 2022 and consummation of the deal in April 2023.

We anticipate that Boston Scientific will be a great fit for Apollo’s portfolio. With their global reach and significant resources in endoscopy-related markets, Boston Scientific is well positioned to increase the adoption of Apollo’s existing products while continuing to invest in additional innovation that is being developed by the Apollo engineers.

**LL: Would you share your thoughts on the future clinical directions of Apollo?**

**Figure 6:**
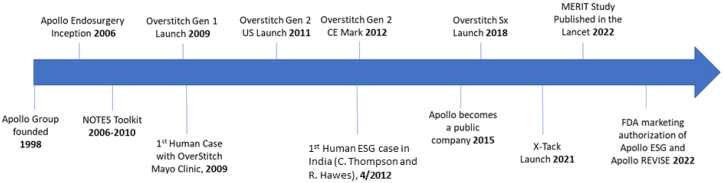
OverStitch and Apollo Endosurgery timeline. *ESG*, Endoscopic sleeve gastroplasty; *FDA*, U.S. Food and Drug Administration; *NOTES*, natural orifice transluminal endoscopic surgery.

**CM:** The dream for Apollo has been to put suturing and closure devices into the hands of every endoscopist. Apollo’s products have enabled endoscopists to expand the range of procedures they perform, and we look forward to extending this to more physicians and into new applications (Fig. 6).

Developing an improved suturing device that is easily used on single-channel endoscopes is one step toward achieving this dream. Later this year, Apollo plans to introduce a next-generation suturing platform designed to significantly enhance the endoluminal suturing experience and usability on single-channel endoscopes. The OverStitch NXT suturing system offers a refined single-channel endoscope suturing product that will be able to address the needs for easier assembly and performance in stressed situations of suturing in retroflexion.

In addition, X-Tack opened the world of defect closure to all shapes and sizes of defects in upper and lower GI locations. Planned refinements in the X-tack system that will allow for greater closure strength and smoother operation, especially in tortuous colons, will position X-Tack as a key device for closure of endoscopic submucosal dissection and EMR defects in high-risk patients.

Our engineers are also developing other new and exciting applications for tools that provide tissue closure and tissue apposition. Many of these are still in an early, confidential stage of development, but I am very excited to see their ideas come to fruition in the coming years.

## Editor’s Closing Remarks

Innovation that leads to disruption of clinical practice is never easy, quick, or linear. This certainly is highlighted by the ultimate success of Apollo Endosurgery in bringing the their endoscopic suturing device to commercial use. It also requires synergy among clinicians, engineers, and investors. A good idea alone is never enough and requires a diverse team, persistence, and some luck. Fortunately, endoscopic suturing is a reality to help many different patient populations with varied issues. However, further simplification and reliability of the suturing device and process would be welcome for staff assisting and physicians using the device. The X-Tack device certainly is a step in the right direction, but further refinements are necessary for any endoscopist to safely and easily perform endoscopic suturing, akin to using through-the-scope clips. Additionally, reimbursement for procedures using these suturing devices remains an ongoing problem. These devices are not cheap and are often poorly reimbursed by insurance companies, if at all. More work is needed to ensure that physicians are appropriately reimbursed for using these devices that can truly help many patients and prevent more expensive surgeries and hospitalizations.

## Disclosure

The following authors disclosed financial relationships: C. McKhann: Consultant for Boston Scientific; full-time employee of Apollo Endosurgery. L. S. Lee: Consultant for 10.13039/100008497*Boston Scientific**, Fractyl, and Fujifilm Medical; research* *support* *from Fujifilm Medical.*
